# Evidence of artefacts made of giant sloth bones in central Brazil around the last glacial maximum

**DOI:** 10.1098/rspb.2023.0316

**Published:** 2023-07-12

**Authors:** Thais R. Pansani, Briana Pobiner, Pierre Gueriau, Mathieu Thoury, Paul Tafforeau, Emmanuel Baranger, Águeda V. Vialou, Denis Vialou, Cormac McSparron, Mariela C. de Castro, Mário A. T. Dantas, Loïc Bertrand, Mírian L. A. F. Pacheco

**Affiliations:** ^1^ Programa de Pós-Graduação em Ecologia e Recursos Naturais, Universidade Federal de São Carlos, São Carlos, São Paulo 13565-905, Brazil; ^2^ Laboratório de Paleobiologia e Astrobiologia, Departamento de Biologia, Universidade Federal de São Carlos, Sorocaba, São Paulo 18052-780, Brazil; ^3^ Université Paris-Saclay, ENS Paris-Saclay, CNRS, PPSM, 91190 Gif-sur-Yvette, France; ^4^ Department of Anthropology, National Museum of Natural History, Smithsonian Institution, Washington, DC 20560, USA; ^5^ Université Paris-Saclay, CNRS, ministère de la Culture, UVSQ, MNHN, Institut photonique d'analyse non-destructive européen des matériaux anciens, 91192 Saint-Aubin, France; ^6^ European Synchrotron Radiation Facility, 38043 Grenoble, France; ^7^ Université Paris-Saclay, ENS Paris-Saclay, CNRS, LMPS, 91190 Gif-sur-Yvette, France; ^8^ Muséum National d'Histoire Naturelle, 75005 Paris, France; ^9^ Universidade de São Paulo, São Paulo 05508-900, Brazil; ^10^ School of Natural and Built Environment, Queen's University Belfast, Belfast, BT9 5AG, UK; ^11^ Laboratório de Biologia Integrativa e Conservação, Departamento de Ciências Biológicas, IBiotec, Universidade Federal de Catalão, 75704-020 Catalão, Goiás, Brazil; ^12^ Laboratório de Ecologia e Geociências, Instituto Multidisciplinar em Saúde, Universidade Federal da Bahia - Campus Anísio Teixeira, 45029-094 Vitória da Conquista, Bahia, Brazil

**Keywords:** zooarchaeology, traceology, South America, Pleistocene, bone surface modification, palaeometry

## Abstract

The peopling of the Americas and human interaction with the Pleistocene megafauna in South America remain hotly debated. The Santa Elina rock shelter in Central Brazil shows evidence of successive human settlements from around the last glacial maximum (LGM) to the Early Holocene. Two Pleistocene archaeological layers include rich lithic industry associated with remains of the extinct giant ground sloth *Glossotherium phoenesis*. The remains include thousands of osteoderms (i.e. dermal bones), three of which were human-modified. In this study, we perform a traceological analysis of these artefacts by optical microscopy, non-destructive scanning electron microscopy, UV/visible photoluminescence and synchrotron-based microtomography. We also describe the spatial association between the giant sloth bone remains and stone tools and provide a Bayesian age model that confirms the timing of this association in two time horizons of the Pleistocene in Santa Elina. The conclusion from our traceological study is that the three giant sloth osteoderms were intentionally modified into artefacts before fossilization of the bones. This provides additional evidence for the contemporaneity of humans and megafauna, and for the human manufacturing of personal artefacts on bone remains of ground sloths, around the LGM in Central Brazil.

## 1. Introduction

### (a) Background

Most Pleistocene megafauna (here defined as mammals with body mass greater than 44 kg [1]) became extinct worldwide by the Pleistocene–Holocene transition. The decline and eventual extinction of these megamammals are generally linked to human impact and climate change [2], but this is a still hotly debated topic for South America [1,3]. Recent studies have raised new perspectives about human arrival in South America around the last glacial maximum (LGM; approx. 19–26 ka) [4], as well as on its impact on the megafauna there (e.g. [5–10]). However, whereas it is currently well accepted that peopling of the Americas happened earlier than the Clovis culture (approx. 13 500 years ago [11]), scepticism about human occupation of the Americas earlier than 16 000 years ago still persists [12]. Questions regarding the timing and routes for human dispersal into the Americas remain open to debate, but human dispersal probably followed multiple routes and time frames, including Pacific coastal and inland (ice-free corridor) routes [13]. In this scenario, Late Pleistocene sites containing evidence of early human occupation in South America should be closely scrutinized with interest.

The Santa Elina rock shelter (Mato Grosso State, Brazil) displays a rock panel rich in paintings, including anthropomorphs (e.g. ‘men with ornaments’), and zoomorphs such as birds, deer, monkeys and tapir. Dated mineral pigments and bonfire structures associated with stone tools and megafaunal remains found in the shelter include Late Pleistocene to the Holocene ages [10,14]. The only Pleistocene megafauna present in the shelter are two extinct giant ground sloths of the species *Glossotherium phoenesis* Cartelle, De Iuliis, Boscaini & Pujos 2019 [15], in two different archaeological layers. Although the bone remains occur in an archaeological context with other material elements of the culture, such as stone tools, they are found in a poor state of preservation, and we could not macroscopically observe any carnivore or cut marks. Along with the giant sloth bones, there are thousands of osteoderms, which are dermal bones once embedded within the skin of the animal [16]. Three osteoderms from the oldest layer of the shelter have previously been reported to have shapes consistent with anthropogenic modification [8,14,17]. However, no deep investigation of these osteoderms has been performed to date, which has led some authors to question the credibility of these bone artefacts (e.g. [18]).

Remains of bone, teeth and shells with human modification for ornamentation purposes may reflect social identity, and are commonly found in archaeological contexts from the Palaeolithic worldwide (e.g. [19–21]). However, these records are rare in the late Quaternary of South America, as the zooarchaeological record for human exploitation of the Pleistocene megafauna in this continent is scarce. Previous publications on Santa Elina have demonstrated the relevance of this archaeological site for documenting human settlement in the continent. Here, we provide information on the spatial and temporal distribution of megafaunal artefacts and bones at Santa Elina and conduct the first detailed macroscopic and microscopic study of the anthropogenically modified osteoderms from this site in order to test the following hypotheses: (i) humans and megafauna cohabitated Central Brazil around the LGM; (ii) humans modified giant sloth osteoderm for cultural purposes other than subsistence. We provide a detailed investigation of the three modified giant sloth osteoderms using advanced non-destructive techniques. Our results offer new insights into megafaunal zooarchaeology in Pleistocene South America and the cultural behaviour of some of the continent's earliest inhabitants.

### (b) Archaeological context

**Figure 1.**
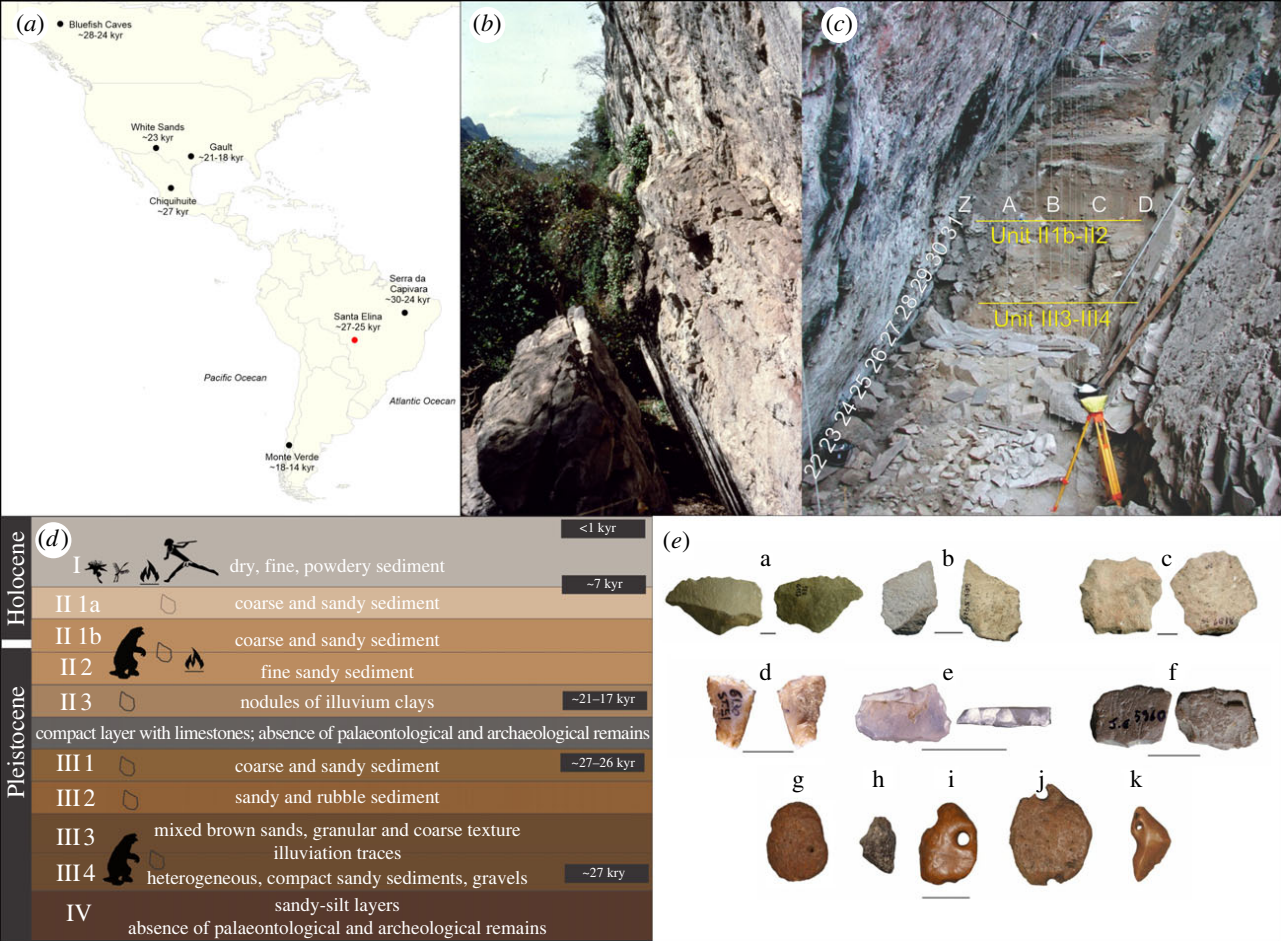
Geological setting and archaeological context of Santa Elina. (*a*) Geographic location of Santa Elina in Brazil (in red), and other selected archaeological sites with evidence for an early human occupation in the Americas (in black [5,6,8,9,22–29]). (*b*) Panoramic view of the rock shelter. (*c*) Excavation area. (*d*) Schematic representation of the archaeological layers at the site, indicating the presence of stone tools, ground sloth remains, fire structures, and wall paintings. Approximate dates based on our Bayesian age model. (*e*) Selected elements found in Unit III4: limestone flakes (a,b) and microblade cores (c) with micro-retouch, retouched siliceous blade cores (d,e), haematite with microwear evidence (f), giant sloth osteoderms (g–k), including unmodified osteoderm (g), possibly burnt bone fragment (*h*) and three osteoderms modified into artefacts (i–k). Scale bars: 1 cm.

Santa Elina rock shelter in Central Brazil (15°27′28′′ S, 56°46′93′′ W) is located in Serra das Araras, a Precambrian setting [10] (figure 1*a,b*). Excavations at the site have revealed successive human occupations, layered in four main units (figure 1*c,d*). Three methods (radiocarbon, uranium–thorium dating (U/Th) and optical stimulated luminescence (OSL) have been used to date bone, charcoal, wood and quartz samples of Santa Elina. Detailed information about the archaeological layers and dating methods used to determine the age of the deposits can be found in previous publications (e.g. [10,30]) and in our electronic supplementary material (electronic supplementary material, text S1). Fossil bones of ground sloths occur only in two layers (Units II1b + II2 and III3 + III4; figure 1*c*), both in clear association with archaeological material, including stone tools and other mineral and rock artefacts. The modified osteoderms discussed here were recovered in Unit III4, at depths of 306, 319 and 323 cm. OSL dating of quartz (*z* = 296 cm) dates this unit to 25.1 ± 2 thousand years ago, whereas U/Th dating of osteoderm (*z* = 310 cm) dates the same unit to 27.0 ± 2 thousand years ago, and radiocarbon dating of microcharcoal from the same depth to 27 402 BP [10]. Unit III is separated from Unit II by a layer of limestone blocks and the absence of fireplace structures, lithics or faunal remains (figure 1*d*). The only difference between Units III1 and III2 to III3 and III4 is based on sediment characteristics and archaeological content; the two latter units bear traces of illuviation [10], but do not present vertical contamination. The rich and diverse archaeological assemblage of Santa Elina includes human-made limestone flakes bearing micro-retouch (figure 1*e*), calcite flakes, and quartz and silex items, some of which could have been used by humans to perform bone surface alterations (figure 1*e*; [10]).

## 2. Results and discussion

### (a) Antiquity and human–megafauna association in Santa Elina

**Figure 2.**
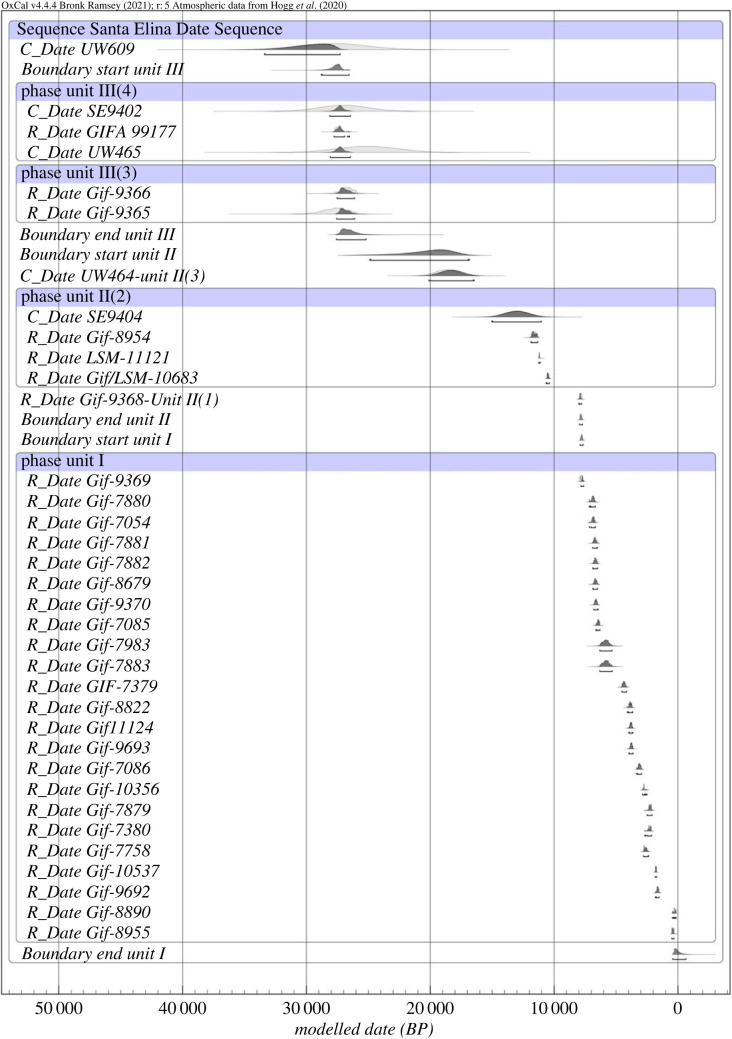
Bayesian age model of Santa Elina.

An OxCal 4.4 was built to calibrate and model the radiocarbon, OLS, and U/Th dates from Santa Elina using the chronological information obtained from the site stratification (see electronic supplementary material, tables S1–S3 and text S2). The acceptable threshold in OxCal4.4 for an agreement index is 60%. The OxCal 4.4 model for Santa Elina has an agreement index of 101.9%, which shows that the stratigraphic model and the dating evidence are compatible with each other (figure 2). The model considers that the units from Santa Elina represent successive stratigraphic phases, with one ending before another begins. Bayesian analysis in OxCal estimates that the human activity at Santa Elina started before the LGM. The beginning of Unit III dates to between 28 743 and 26 536 BP (95.4% probability), with Unit II commencing some time between 24 922 and 16 880 BP (95.4% probability), probably between 21 473 and 17 778 BP (68.3% probability) and extending through the end of the Pleistocene into the Holocene, ending between 7947 and 7714 BP (95.4% probability). Deposition of Unit I started between 7901 and 7656 BP, and possibly continued into the modern era with two samples dated as recent, which are probably only a very few centuries old (see electronic supplementary material, text S3, tables S2–S3). Two- and three-dimensional georeferenced maps of archaeological materials from Santa Elina support the evidence for human activity, in the form of stone tools and other lithic artefacts, associated with remains of *G. phoenesis* in the two distinct periods of occupation during the Late Pleistocene (see electronic supplementary material, text S4 and figure S3). Through the two-dimensional spatial distribution of the osteoderms, it is possible to observe unusual accumulations of osteoderms in specific loci that might be related to intentional disposal (greater than 1000 elements per square metre; electronic supplementary material, figure S4). However, more efforts are needed to elucidate the taphonomic history of Santa Elina, including studies of the weathering stages of the ground sloth bones, which has not yet been undertaken. The meticulous investigation of the sedimentary context of Santa Elina also demands further efforts.

### (b) Human and rodent modification of giant sloth osteoderms

**Figure 3.**
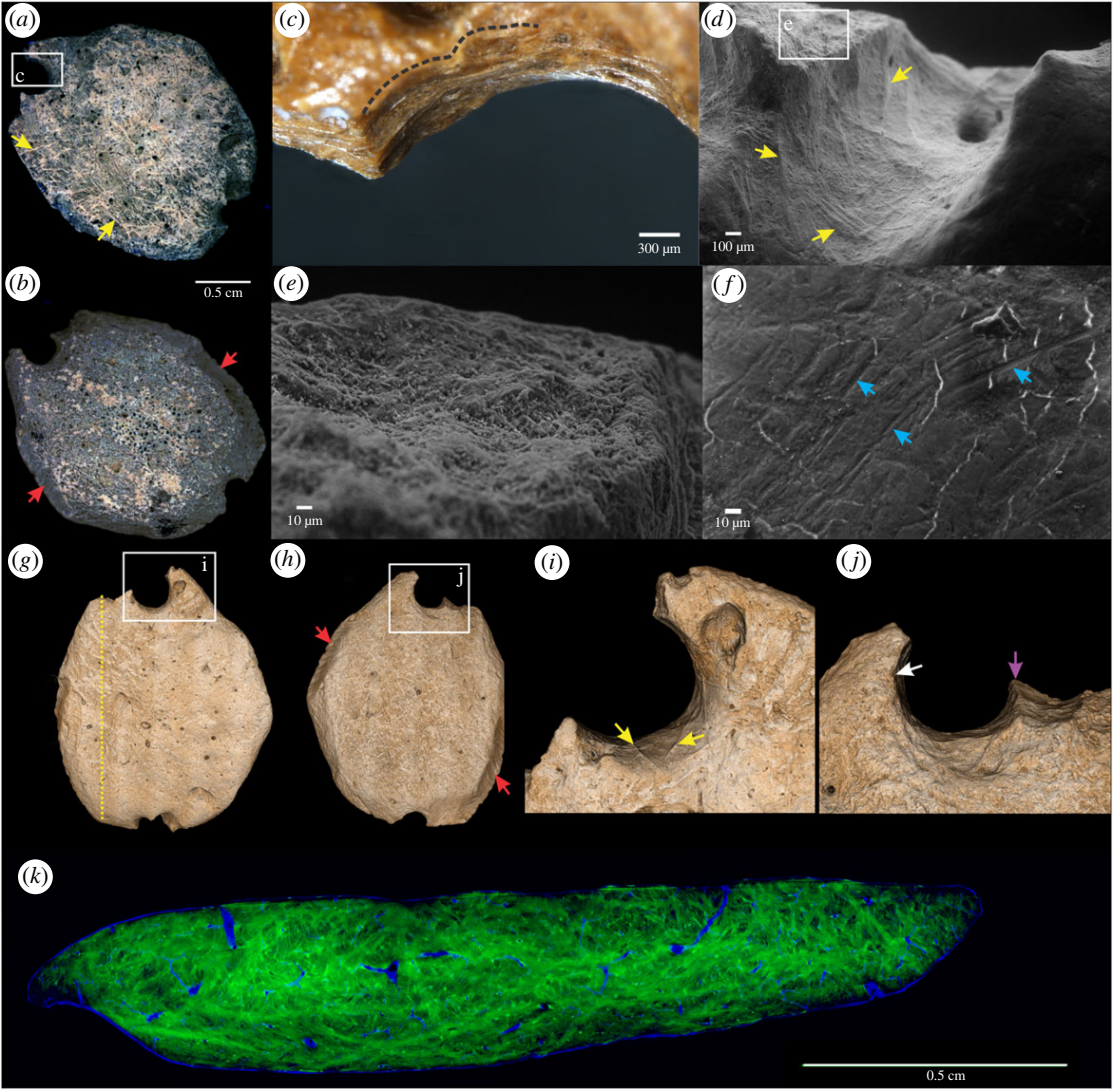
Anthropically modified giant sloth osteoderm (SEI6059). (*a,b*) Macroscopic view using photoluminescence imaging, side 1 and 2, respectively. (*c*) Microscopic and (*d*) scanning electron microscopy images of a hole drilled into the osteoderm (zoomed broken perforation area in *c*; notice drilling traces and smooth deformation). (*e,f*) SEM images. (*e*) Fracture surface exposing internal bone tissue. (*f*) Scraping marks on the polished surface. (*g–j*) SR-µCT reconstructed images; see main text for discussion. (*k*) False-colour image of the virtual cross section SR-µCT along the yellow dotted line in *g* highlighting collagen fibre bundles (green) and vascular channels (blue). In all figures: yellow arrows point to fibre bundles exposed by intentional and intensive polishing or extensive use-wear, red arrows to sets of likely rodent gnawing, blue arrows to scraping marks, white arrow to deformation of the perforation wall probably due to use-wear, and purple arrow to the well-delimited wall of the broken deliberate perforation.

**Figure 4.**
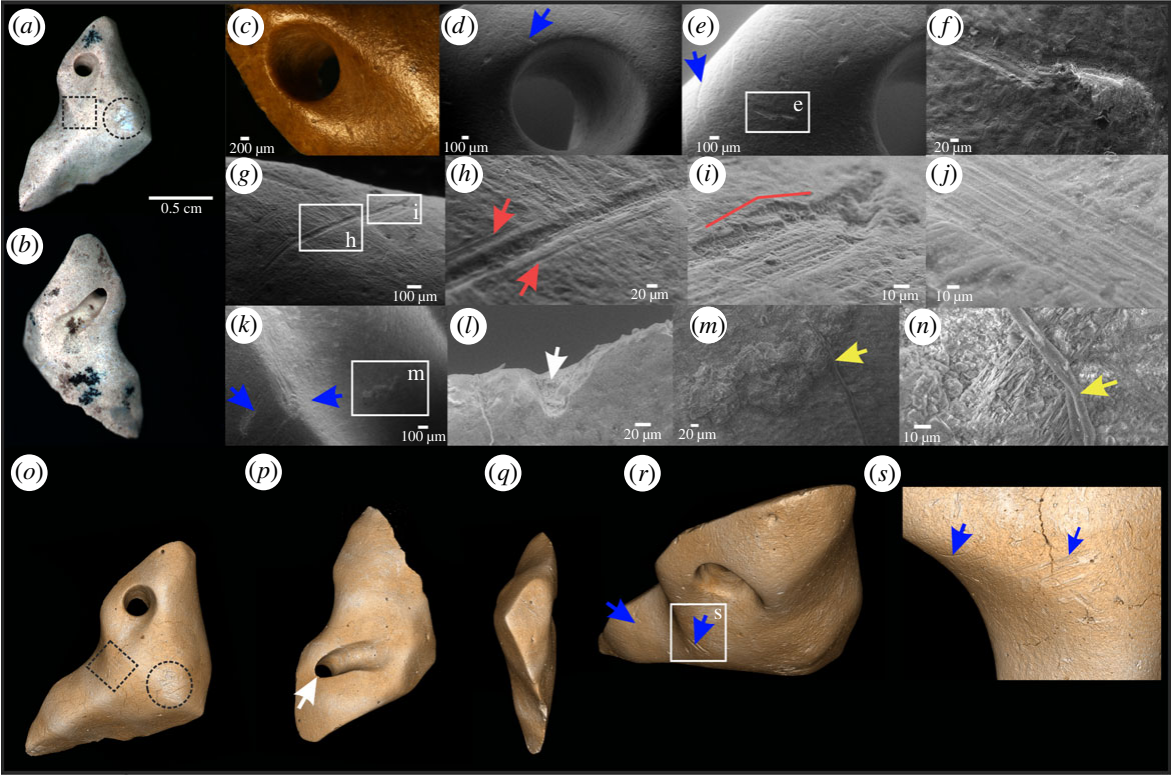
Anthropically modified giant sloth osteoderm (SEI6557). (*a,b*) Macroscopic full-field photoluminescence images. Notice human-manufactured curvature and retouched edges showing intentional shaping of the bone, sides 1 and 2, respectively. Notice the uniform PL contrast along the bone. An area of possible trampling damage with distinctive brighter PL contrast (black dotted circle), can be compared with an area where ancient marks are uniform to the bone surface and perforation (black dotted square; see detailed morphology in the three-dimensional image in *o*). Incisions and other multiple fine parallel striations from scraping over a broad area are frequent along this osteoderm. (*c*) Notice polish aspect and worn and deformed perforation, probably the result of use-wear of the attached system. (*d–n*) Scanning electron microscopy images. Blue arrows indicate several scars around the drilled hole on side 1 (*d, e*), and side 2 (*k*). (*g*) Elongated and deep curve-shaped groove with regular internal microstriations located in a concave area of the bone. Shoulder effect is indicated by red arrows (*h*), and Hertzian cone highlighted in the red line (*i*). (*j*) Straight-walled scrape mark with multiple internal striations. (*l*) One of the micro-breakages present on the top of the enlarged perforation on side 2 (*b, p*). Notice the exposure of internal bone tissue indicated by the white arrow. (*m,n*) Yellow arrows indicate probable collagen fibre residue, trapped in a translucent gelatinous matrix inside the concave hole area of side 2 (zoom in *m* and *n*). (*o–s*) Three-dimensional SR-µCT images. Blue arrows indicate parallel deep marks in curvature areas, unlikely to be produced by trampling.

**Figure 5.**
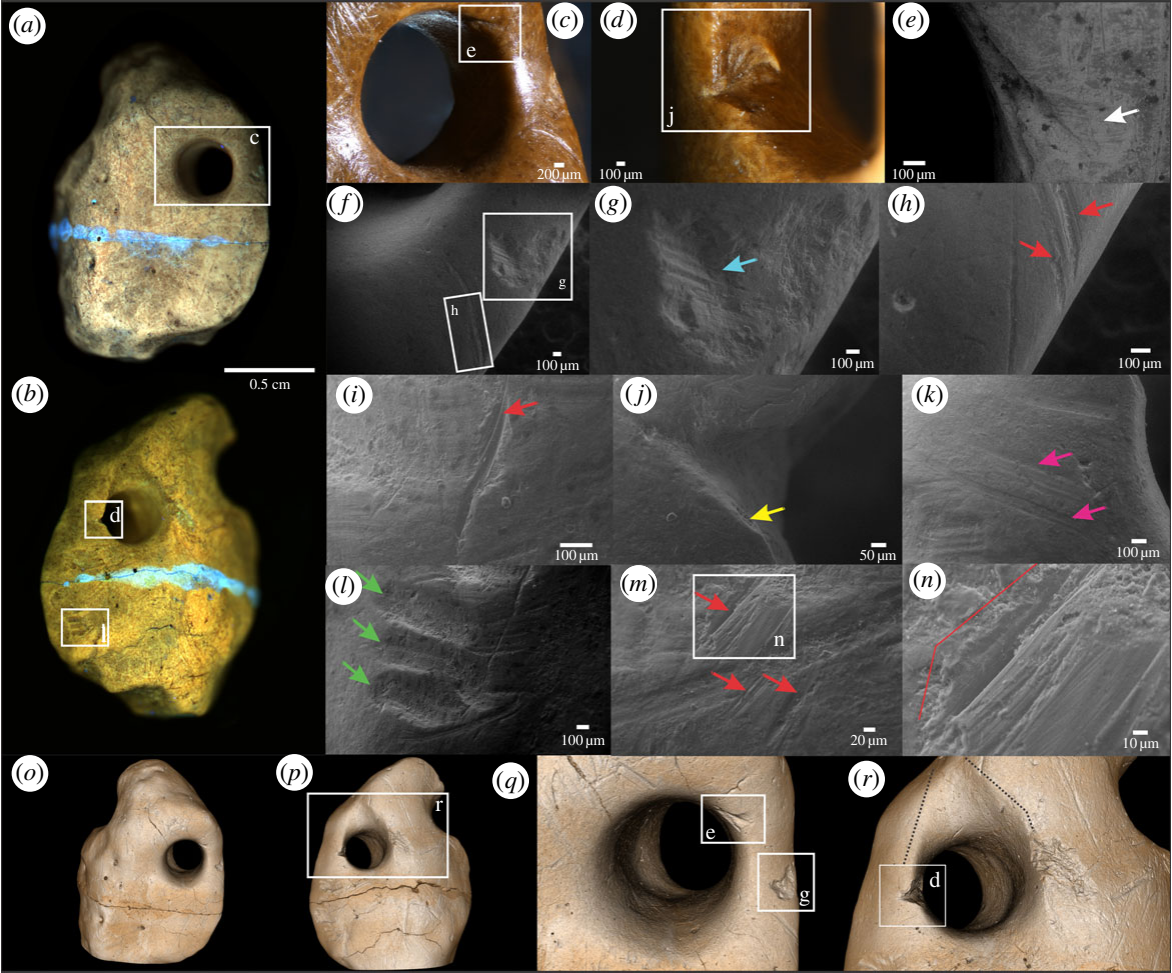
Anthropically modified giant sloth osteoderm (SEI6386). (*a,b*) Macroscopic photoluminescence images, sides 1 and 2, respectively. Blueish horizontal line across the sample corresponds to glued-area. (*c,d*) Microscopic images. Notice use-wear traces around the perforation hole on side 1 (*c*), and human modification scar on the lateral of the perforation hole on side 2 (*d*). (*e–n*) Scanning electron microscopy images, (*e*) AsB detector, (*f–n*) VPSE detector. (*d, j*) Anthropic scar, probably accidentally produced by stone tools during the process of drilling the hole. (*e*) V-shaped scar incision, also probably produced during the drilling of the hole. White arrows indicate ‘barbs’ features on the terminal of the stone tool incision. (*f, h*) Two elongated incision marks (red arrows), following the curvature of the bone, with internal microstriations and shoulder effects, and accompanied by a single mark (probably trampling). (*g*) Percussion notch associated with internal microstriation (blue arrow), probably from slippage. (*i*) Single incision mark (red arrows) accompanied by Hertzian cone formations. (*j*) SEM image of stone tool-inflicted scar. Yellow arrow indicates exposure of periosteum. (*k*) Scraping marks lateral to the perforation hole (pink arrows). (*l*) Small deep grooves, probably resulting from repeated gnawing in the same area made by a small rodent (green arrows). (*m*) Incisions probably inflicted by a series of single strokes made by a stone tool (red arrows), overlapping a linear mark. (*n*) Zoomed image in which a red delimitation indicates the area of flaking. (*o–r*) SR-µCT images highlight natural bone structures, such as small foramen, and anthropogenically caused modifications, including the smoothness of circular depressions, on both sides of the osteoderm and around the perforated hole. (*r*) Use-wear deformation in V-shape above the hole (black dotted lines), probably resulting from the suspension of the object or interaction with string.

Here we provide evidence that three giant ground sloth osteoderms from Unit III4 of Santa Elina (SEI6059, SEI6557 and SEI6386) were anthropogenically modified. The combination of different magnification and imaging techniques allows observation of the polished appearance and use-wear traces around the bone surfaces and hole perforations. The presence of several marks from human modification, including drilled perforations, polishing, multi-directional scratches and use-wear traces (figures 3–5; electronic supplementary material, figures S11–S13) suggests their anthropic nature and extensive use. We document smoothing of the surface; traces of stone tool interaction with bone, including incisions and scars, scraping marks, scratches, percussion notches; polish and gloss; use-wear smoothing of the rim and the attachment systems; and animal-inflicted modifications on the three osteoderms (figures 3–5; electronic supplementary material, figures S11–S13). These observations show that these three osteoderms were modified by humans into artefacts, probably personal ornaments. Two osteoderms (SEI6557 and SEI6386) present a circular perforation that goes through the bone, with a well-defined and regular rim and scars along the edges indicating a deliberate human manufacturing process. One osteoderm (SEI6059) has two broken holes on its borders; this osteoderm appears polished and flattened on its whole surface and has been modified and shaped to be thinner than others with one side more polished and smoothed than the other, and one hole larger than the other in opposite and symmetric portions of the bone (figure 3; electronic supplementary material, figure S13). Osteoderm SEI6386 presents a remarkable concave area on one of the sides of its perforation hole. Osteoderm SEI6557 presents a similar pattern. We attribute these deformations to use-wear, possibly due to string interaction or suspension in an attachment system. Osteoderm SEI6557 has a unique shape, with the hole perforation connected to an elongated structure that we interpret as deformed ancient grooves of the natural osteoderm. These grooves may naturally intersect with foramina on both sides of the bone (see microscopic images of FR3B in electronic supplementary material, figure S5). The morphological difference between natural foramina and intentionally perforated holes can be seen both macroscopically and microscopically (electronic supplementary material, figures S5–S8).

Unmodified mylodontid osteoderms show a naturally rough external surface [16], notably different from the smooth polished surfaces of the three human-modified osteoderms. Among the thousands of fossil osteoderms on the site, the perforated and polished state of the three osteoderms studied here is exceptional (see electronic supplementary material, text S5, table S11). Superficial pits and vascular foramina are present on the external surface of some, but not all, osteoderms of *G. phoenesis* from Santa Elina. When present, they penetrate the bone tissue and appear significantly smaller than the human perforated holes (see electronic supplementary material, figures S5–S6 and table S12). It is possible that the natural foramina may have been used as a starting point for the perforation process operated by humans. Analyses of the perforation morphology of the larger holes on these three modified osteoderms allow us to exclude natural processes as possible agents of modification. Bioerosion traces generally produce shallow circular holes and chambers with eroded morphology and deformed or coarse rims, and mainly reach the internal spongy structure [31]. These bioerosion traces are significantly different from the smooth, well-delimited rim of the perforations described here (see electronic supplementary material, text S5). Armadillo osteoderms perforated by flea parasites present a conic section of their irregularly shaped hole morphology, and have a heterogeneous and corroded appearance [32]. The osteoderm artefacts from Santa Elina also do not present a cracked surface and ‘torn-like’ appearance resulting from digestion or regurgitation [33]. We also note that osteoderm SEI6059 presents one side more polished than the other (which is not expected in the case of a digestion product). In addition, the well-preserved and distinguishable features of scraping and incision marks would probably have been erased with acidic corrosion, as can occur with cut marks on bones reported in previous studies [34,35]. Thus, we reject the possibility of natural or non-human causes of modification of these osteoderms.

The human-made perforation holes in osteoderms SEI6557 and SEI6386 are polished and worn (figures 4 and 5; electronic supplementary material, figures S11–S12). While it is still possible to identify remaining traces from scraping and intentional drilling, they are in general extremely worn, which has erased most of the rough internal damage that was probably induced by the perforation process. This smoothing could be explained by the osteoderms being suspended as pendants, or a substantially long period of use (e.g. contact with solid or softer organic material, other ornament beads, clothes, skin, etc.), which has been documented in previous experiments [36] and for other ancient ornaments (e.g. [37–40]). Still, the use-wear traces are visible. The holes and marks exhibit the same homogeneous colour as the rest of the osteoderms' outer surfaces (figures 3–5; electronic supplementary material, figures S11–S13), suggesting that the modifications occurred prior to the final burial of the osteoderms, rather than being created afterwards on already fossilized bones [41].

Bone damage and human modification marks on the surface of highly modified osteoderms were studied in detail from three-dimensional reconstructions generated from synchrotron-radiation-based micro-computed tomography (SR-µCT; figures 3*g–j*, 4*o–s*, 5*o–r*, and electronic supplementary material, videos). Direct visualization of the retouched and sharp edges in osteoderm SEI6557, and intensive deformation of the rim evidenced by the concave surface in osteoderm SEI6386 (figure 4*r*), are possible on the three-dimensional volumes of SR-µCT, overcoming the limitations of conventional surface profilometry. The concave use-wear traces observed in osteoderms SEI6386 and SEI6557, although less intense in the latter, may be attributed to interaction with strings, clothes or pressure against other pieces [37–39]. The double perforated osteoderm (SEI6059) and one of the single perforated osteoderms (SEI6386) were found in a broken state, which may be related to having been lost or discarded after being worn [42]. Indeed, experiments show that even within only months, attached ornaments can become worn, deformed and broken (e.g. [43]). All the characteristics meticulously observed in the three modified osteoderms from Santa Elina suggest that they were used as ornaments. However, further technological and ethnographical studies of these artefacts are encouraged to allow a more precise interpretation of their exact function.

We performed a zooarchaeological experiment and a subsequent comparative study between human modifications made on a modern dry osteoderm (giant armadillo *Priodontes maximus,* UFCAT-MAM 1/6), and on a fossil osteoderm (*G. phoenesis*, SEI8004), and compared them with two of the modified fossil osteoderms from Santa Elina (SEI6557 and SEI6386). We observed similar characteristics of the stone tool marks made on the modern armadillo bone and the osteoderm artefacts, although the latter have a lower quality of preservation. These characteristics includes intensive flaking and Hertzian cones made on the shoulders of the stone tool marks and the observation of blood vessel impressions, characterized as funnel-like openings with rounded edges [44], as well as isolated collagen fibre whose morphology is highly similar to the residue material preserved in the osteoderm SEI6557 (figure 4*m,n*). By contrast, these features are not observed on our experimentally modified fossil osteoderm (electronic supplementary material, figure S15).

**Figure 6.**
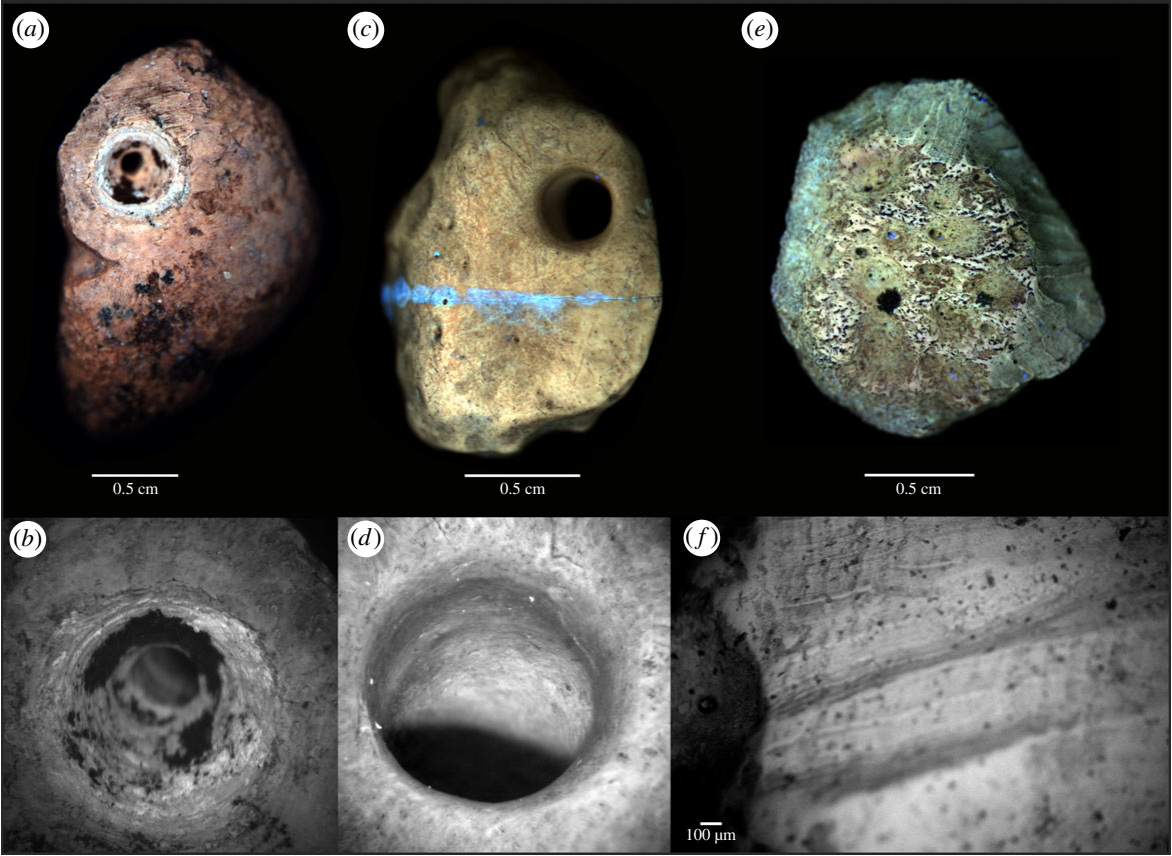
Comparison of modification between an experimentally modified fossil osteoderm, one of the tree artefacts, and a rodent gnawed osteoderm. (*a*) PL image of experimentally modified osteoderm SEI8004 and (*b*) microscopic view of its perforated hole. (*c*) PL image of the artefact SEI6386 and (*d*) microscopic view of its perforated hole. (*e*) PL image of a rodent gnawed osteoderm and (*f*) SEM image of a set of their gnawing marks (AsB detector). Notice the brighter and grooved hole in the experimentally modified osteoderm and the worn rim perforation in the ancient anthropically modified osteoderm. Blue contrast in the osteoderm SEI6386 (*c*) corresponds to a glued area. Notice oxides overlapping internal microstrations of the ancient gnawing marks; similar aspects are found on the anthropically modified osteoderms from Santa Elina. Notice the uniform luminescence pattern of the gnawed bone, which is similar to the PL behaviour observed in the anthropic modified osteoderms in this study.

We tested another, rarely exploited property of fossil bone materials: the UV/visible photoluminescence (PL) response of their constitutive minerals. The composition of altered apatite minerals such as those found in bones and soft tissue remains (e.g. fossilized collagen and muscle fibres) is modified during weathering or diagenesis. Substitutions by elements from percolating fluids (e.g. by rare earth elements, F, Mn, Fe or Sr), as well as structural defects formed in the crystallites, can be mapped and studied by photoluminescence (see [45,46] and references therein). While biological apatites generally show a weak PL signal, these defects can result in intense and specific PL emissions in ancient apatites. PL contrasts in a sample can be tested to recover information about its taphonomic history. False colour PL images (see *Material and methods*) highlight the differences between osteoderms (figure 6). To further explore the contemporaneity of the perforation, we experimentally modified an ancient osteoderm (SEI8004, perforated and polished by our team). PL shows a high contrast between the low luminescent osteoderm surface and the new perforation, which emits strongly at 514 ± 15 nm under excitation at 385 nm. This demonstrates a significant difference between the chemical composition of the patina formed over a very long period of time and the subsurface revealed by experimental modification. By contrast, the two modified osteoderms SEI6557 (figure 4*a,b*) and SEI6386 (figure 5*a,b*) show uniform luminescence along the hole and anthropogenic marks with little or no detectable difference in hue. In SEI6386, the only variation observed in the PL signal occurs in the area consolidated with glue. These observations indicate that, while recent human perforations expose a less altered internal bone structure and mineralogy than the osteoderm surface, anthropogenically modified osteoderms display a homogeneous composition of surface defects. This suggests a homogeneous history of the surface of anthropogenically modified osteoderms, i.e. ancient modifications and marks that were made before fossilization, most likely before burial, while the osteoderms were in fresh or dry states (according to the definitions in [47]). Most interestingly, PL imaging of the flat, smooth human-modified osteoderm SEI6059 reveals that its most intensively scraped and polished flat surface exposes histological features, such as the networks of over-crossing mineralized fibre bundles in variable orientation and vascular foramina pits (figure 3; electronic supplementary material, figure S13M). This is direct evidence that human intervention and ancient use of this osteoderm abraded the osteoderm material down to deep histological levels, the detailed morphology of which has been preserved to this day.

Traces of non-human ecological interaction, such as rodent gnawing marks, are commonly found on osteoderms from Santa Elina. These marks are visible even macroscopically along the edges of osteoderm FR10 from Santa Elina, in two sets along the edges of the artefact SEI6059 (figure 3*b*,*h*), and on the surface of artefact SEI6386 (figure 5*l*). Broad, shallow, flat-bottomed, elongated parallel pairs of grooves of rodent gnawing, consistent in size and shape along the bone edges, also exhibiting internal but shallow microstriations, were observed in osteoderm FR10 using macroscopic and microscopic visualization with SEM (electronic supplementary material, figure S7E-F). Repeated rodent gnawing in a restricted area may result in deep and wider grooves ([48]; figures 3*h* and 5*l*). The presence of morphological features, such as foramina pits and natural depressions, surrounded by rodent gnawing marks, led to a uniform luminescence in PL imaging in FR10 (electronic supplementary material, figure S7E). Rodents tend to gnaw on carcasses in fresh or dry state, mainly to obtain lipid or minerals from the bone and wear their teeth [49]. Rodent gnawing happens while bones are still exposed on the ground surface, before the final burial and fossilization of bones [50]. The rodent gnawing on the edges of a flat osteoderm (SEI6059) and surface of osteoderm SEI6386 indicates pre-burial modification of the bones. The uniform pattern observed using PL supports the hypothesis that osteoderm FR10 was gnawed by rodents in a fresh or dry state (pre-burial) and corroborates our interpretation that any human-induced modification was made pre-burial as it led to similar PL behaviour. The PL behaviour in osteoderm SEI6386 (figures 5*a,b* and 6*c*) revealed a uniform chemical contrast in the hole perforation, rodent marks (figure 5*l*) and other marks here identified as anthropogenic modifications. The uniform chemical contrast observed in the anthropic marks, hole perforation and mineralized bone surface of osteoderm SEI6557 provides a similar interpretation (figure 4*a,b*).

Although rodents can gnaw fresh bones and bones with several years of subaerial exposure [50], we reject the hypothesis of reburial, that is, the osteoderms were not dug up, polished and buried again. The evidence for this is that the ground sloth's bones and hundreds of osteoderms are in the same depositional context. In addition, the giant sloth skeleton is in an archaeological context with other cultural material, such as stone tools (electronic supplementary material, figures S2–S4). If the osteoderms were not polished while fresh, they were at least polished as dry remains, which is different from being polished after they were mineralized by fossilization. The human modification happened before burial and, therefore, diagenesis, indicating a temporal proximity between the human occupants of the Santa Elina shelter and the megafauna during Unit III4 (LGM boundary). Therefore, our strongest hypothesis for the sequence of events is: osteoderm modification (by humans), followed by rodent gnawing and other ecological interactions, followed by burial.

## 3. Final remarks

Detailed imaging and traceological analyses on three anthropogenically modified osteoderms of the giant ground sloth from layers dated to around the LGM in Santa Elina show direct evidence for human modification of these bones. The different techniques and magnifications applied in this traceological study, as well as the combination of the evidence analysed (the morphology of the perforation holes and bone surface modifications, the environmental/archaeological context with a rich lithic industry, and the absence of other large faunal remains such as carnivores that might have inflicted bone surface modifications) all support our identification of human modification of three giant sloth osteoderms in a pre-burial context. These are, so far, the only record of presumably personal artefacts from the LGM in the Americas. Their unique and diverse shapes, hole perforation sizes and presence of diverse anthropogenic traces suggest that different tools and techniques could have been employed during the production and finishing process of the final artefacts. The worn hole perforations and deformed surfaces, as well as attachment systems and use-wear traces, suggest their extensive use, probably as suspended ornaments. Their rarity (three artefacts among thousands of osteoderms) and the broken condition of two of them suggest that they may have been lost or intentionally discarded because of breakage. This rarity can also be explained as personal items are generally taken along when people leave their settlements [51].

Based on the zooarchaeological context of Santa Elina, we conclude that the most parsimonious scenario is that humans collected and modified the osteoderms of the ground sloth exposed on the shelter floor, used the artefacts during their occupations and/or uses of the shelter, and subsequently lost or discarded the artefacts in the shelter. However, we recognize the possibility that these three osteoderms could have been obtained and modified from a different ground sloth individual elsewhere, transported to Santa Elina, and then lost or discarded there. In this scenario, the human modification of these three osteoderms would bear no relationship with the ground sloth skeleton found in Unit III4 of Santa Elina. Yet, the osteoderms are still in archaeological context with other elements of material culture. This scenario would still have ramifications regarding the culture and behaviour of early human populations of the region. The movement of cultural artefacts over long distances suggests potential exchange networks and a possible symbolic value of these objects in their communities [52,53]. We cannot evaluate whether this alternative scenario is accurate for the three modified osteoderms found at Santa Elina with the evidence we provide in this study. Additional evidence and research, such as use-wear and residue analyses on the stone tools from Unit III4, still absent for Santa Elina, would be needed to elucidate if the production of these bone artefacts was undertaken locally.

Santa Elina challenges mainstream claims on peopling of the Americas, in favour of a model in which people first reached out to the American continent during, or even earlier than, the LGM. It agrees with evidence reported from other sites that suggests early human presence in North America, such as the Bluefish Caves in Canada [22,23], the White Sands National Park in NM, USA [24,25], the Gault site in TX, USA [26], the Hartly mammoth locality in NM, USA [54], the Chiquihuite Cave in Mexico [27], which has retouched artefacts similar to the ones found in Unit III of Santa Elina [55]; and in South America, such as several localities at the Serra da Capivara National Park in northeast Brazil [5,6,9,28,29], Monte Verde II in Chile [8], and those with claims for human–megafauna interaction, such as El Muaco and Taima–Taima in Venezuela [56], and Arroyo del Vizcaíno in Uruguay [6] (although the pre-LGM human presence in the latter has been disputed [57]). The Cerutti Mastodon site in CA, USA, stands out as an even more controversial site which has been suggested to present evidence for human presence and megafauna butchery during an interglacial period (approx. 130 000 BP; [58]), but this evidence has been strongly contested by several authors (e.g. [59,60]). Whereas some authors question the veracity of pre-LGM human settlements in the Americas (e.g. [61,62]), others interpret that the scarcity of archaeological evidence of pre-LGM sites in the Americas may be explained by an initial settlement occurring earlier than the introduction of elaborate stone technology in the continent [54], and that pre-LGM hunter/gatherers populations were probably affected by climate pressures and remained at low densities until their wide dispersal after the deglaciation of the Cordilleran and Laurentide ice sheets [63].

The evidence presented here of anthropic modification of giant sloth osteoderms during the early LGM at Santa Elina supports the hypothesis that humans were in South America thousands of years before the extinction of the Pleistocene megafauna in the continent. Together with the presence of another giant sloth individual in a more recent level of the site (Unit II2), this evidence might suggest that the human presence in South America was not the main agent responsible for the megafauna extinction. It agrees with previous research claiming that it would have taken thousands of years for hunter–gatherer populations to expand and dominate this vast and diverse continent [63]. Further investigations, including a detailed taphonomic study of the ground sloth bone assemblage from Santa Elina, could add to this discussion.

Our contribution reinforces Santa Elina as a pivotal site in the debate on human occupation, symbolic behaviour and megafaunal bone modification in the Late Pleistocene of South America. The significance of the evidence from this rock shelter can be summarized as follows: (i) the archaeological context and association of giant sloth remains with cultural elements from human occupations in two periods of the Late Pleistocene [10]; (ii) the anthropic modification of giant sloth osteoderms from a layer dated to the early LGM, evidenced by intentional shaping and perforation of these bones; (iii) the interpretation of these modified osteoderms as artefacts, probably used as personal ornaments, based on use-wear traces and smoothing deformation; and (iv) evidence that these modifications were performed prior to the burial and fossilization of the giant sloth osteoderms, during the last glacial period.

## 4. Material and methods

### (a) Bayesian age model

Thirty-five radiocarbon, OSL and U/Th dates from Santa Elina, spread through four stratigraphic units (I–IV) (electronic supplementary material, table S1), were modelled and calibrated with OxCal 4.4, using the SHCal20 calibration curve [64] (see electronic supplementary material, text S2). The clear and well-understood stratigraphy of Santa Elina [10] allows the model of this site to be easily created in OxCal. OxCal cannot *prove* that a model is correct, as more than one possible model can fit the data, but it can reject models which are incompatible with the radiocarbon evidence. In addition to being able to tell which models are compatible with the radiocarbon evidence, OxCal has a number of tools for estimating the start, end or span of defined phases (electronic supplementary material, text S2).

### (b) Taphonomic aspects of giant sloth osteoderms

We provide for comparison images of the morphology of 19 giant sloth osteoderms from Santa Elina without anthropic marks (electronic supplementary material, figure S5–S6). We compare the three fossil osteoderms that we confirm as human-modified (osteoderms SEI6059, SEI6557 and SEI6386) with one natural osteoderm (specimen FR3A), one osteoderm exhibiting rodent tooth marks (specimen FR10), one osteoderm with bioerosion damage (specimen FR6), one fossil osteoderm experimentally modified into an ornament (specimen SEI8004) and one modern armadillo osteoderm experimentally modified (specimen UFCAT-MAM 1/6; see details in electronic supplementary material, texts S5–S6), at micro- and macroscales using scanning electron microscopy (SEM), photoluminescence (PL) multispectral imaging, and synchrotron-based X-ray microtomography (SR-µCT). Identification of the agent of bone surface modifications (e.g. bioerosion, rodent gnawing, trampling marks, anthropogenic marks and use-wear traces) relied on established signature criteria following previous research (e.g. [37,48,49,65–67]).

Microscopic images were taken using optical microscopes (Leica), a stereomicroscope Nikon SMZ-25, and a scanning electron microscope. SEM images were collected using two microscopes: (i) low vacuum (approx. 50–70 Pa) with an acceleration voltage of 15 kV, using the Zeiss supra 55VP FEG-SEM at the IPANEMA laboratory, with a variable pressure secondary electron detector (VPSE, default) or an angle selective beam detector (AsB, where noted); and (ii) low vacuum with an acceleration voltage of 15 kV using the TM3000 tabletop microscope Hitachi at the Laboratório de Pesquisa em Bioenergia e Materiais Lignocelulósicos, UFSCar.

PL multispectral macro- and micro-imaging were performed using a prototype set-up developed by IPANEMA, allowing for the collection of reflection and luminescence images in the UV, visible and near-infrared spectral ranges [68]. We collected luminescence emissions in the blue (472 nm), green (514 nm), yellow (571 nm) and red (685 nm) domains under UV (385 nm) illumination. The resulting greyscale images were combined into false colour RGB images using ImageJ. False colour RGB images presented here were produced using two settings: red, emission at 685 ± 20 nm; green, emission at 571 ± 36 nm; blue, emission at 514 ± 15 nm for SEI6557, SEI6386 and SEI8004 (figures 4*a,b*, 5*a,b*, 6*a,c*), and red, emission at 685 ± 20 nm; green, emission at 571 ± 36 nm; blue, emission at 472 ± 15 nm for SEI6059, FR10 and FR3A (figures 3*a,b* and 6*e*; electronic supplementary material, figure S6B).

Synchrotron-based X-ray µCT was performed on the three modified fossil osteoderms at the BM05 beamline of the European Synchrotron Radiation Facility synchrotron using a polychromatic beam with a detected average energy of 75 keV. This energy was obtained by filtering the white beam from the 0.85 T dipole wiggler source with 50 mm of SiO_2_ (as a series of 10 bars of 5 mm of diameter used to homogenize vertically the beam power profile) and 2.3 mm Al. Scans were performed using 6000 projections over 360° using a detector configuration giving a pixel size of 3.9 µm (500 µm thick LuAg:Ce scintillator coupled to a PCO edge 4.2 CLHS sCMOS camera using a zoom optic based on a Canon 65 mm MP-E f/2.8 supermacro objective). The sample-to-detector distance was fixed at 1.4 m in order to have propagation phase contrast effect to reveal fine internal and external details. The available field of view was extended horizontally by positioning the rotation axis off-centre and extended vertically by recording a series of acquisitions with vertical movement of the sample. The volume (3.9 µm isotropic voxel size) was reconstructed from the radiographs by using a filtered back-projection algorithm implemented in PyHST2 software [69], with a single distance phase retrieval algorithm [70]. Three-dimensional rendering was performed using 3DSlicer (https://www.slicer.org/). We generated a false colour overlay of the median and standard deviation projections of 40 tomograms using ImageJ, displayed as levels of green and blue, respectively (figure 3*k*).

## Data Availability

The data are provided in electronic supplementary material [71].
